# The importance of hair in human perception of electric fields – A double-blind repeated measures study

**DOI:** 10.1038/s41598-026-52898-6

**Published:** 2026-05-13

**Authors:** Kathrin Jankowiak, Andrea Kaifie, Julia Krabbe, Maike F. Dohrn, Simon Kimpeler, Frederik Mingers, Ralph Kühn, Thomas Kraus, Michael Kursawe

**Affiliations:** 1 Research Center for Bioelectromagnetic Interaction (femu), Institute for Occupational, Social and Environmental Medicine, Uniklinik RWTH Aachen University, Pauwelsstraße 30, 52074 Aachen, Germany; 2 Institute for Occupational, Social and Environmental Medicine, Uniklinik RWTH Aachen University, Aachen, Germany; 3 Department of Neurology, Uniklinik RWTH Aachen University, Aachen, Germany; 4 Institute for High Voltage Equipment and Grids, Digitalization and Power Economics, RWTH Aachen University, Aachen, Germany

**Keywords:** Electric Field, Exposure, Perception, Human hair, Environmental factors, Environmental social sciences, Neuroscience, Psychology, Psychology

## Abstract

Electric fields (EFs) are an integral part of modern life which might affect humans in terms of conscious perception. Despite their ubiquity, the mechanisms underlying EF perception remain underexplored. To elucidate interindividual variance, this study investigates the influence of human hair on the perception of alternating current (AC) EFs, direct current (DC) EFs, and hybrid EFs using a double-blind repeated measures design. Thirty healthy participants were exposed to various EF strengths while their hair characteristics and environmental conditions were systematically manipulated. The findings indicate that hair plays a crucial role in EF detection, as removal of hair markedly increased detection thresholds for all EF types. Furthermore, correlations were observed between hair moisture content and DC EF sensitivity, as well as between arm hair roughness and AC EF sensitivity. Factors such as environmental relative humidity and the application of mascara directly influenced the EF detection performance. The findings suggest that both the presence and properties of hair notably contribute to how humans are affected in their perceptual experience of EFs, highlighting the need for further research into individual characteristics that influence the EF perception mechanisms.

## Background

Electric fields (EFs) are already an integral part of our everyday life. Nevertheless, the human perception of EFs has been investigated in only a few studies so far. However, it has been clearly shown that humans are able to consciously perceive both static EFs and low-frequency EFs. Scientists tested the perceptibility of direct current (DC) EFs, alternating current (AC) EFs and the combination of both EF types (hybrid EFs). Although human static and low-frequency EF perceptibility is well documented, underlying biological mechanisms are not completely understood.

Local DC exposure on the arm was detected by the participants at an average of 375 kV/m^1^, while local AC EFs between 8 and 33 kV/m were perceived^2^. In these studies, the participants indicated verbally^1^ or by pressing a button^2^ whether they had a perception in the arm area. Participants perceived average EF strengths of 25 kV/m DC and 15 kV/m AC under high-voltage power lines in the open field^3^. The participants were interviewed verbally and asked to indicate both perceptibility and qualitative assessment on a 6-point scale. Other studies conducted under laboratory conditions showed significantly lower detection thresholds under whole-body exposure than under local exposure, especially in the DC condition^4–6^. One explanation for these results could be the field elevation at the body part closest to the source, which means that EFs under whole-body exposure could already be perceived at lower EF strengths^7^.

In an experimental study, Blondin et al.^4^ investigated the human perception of static EFs. In this study, 48 participants were exposed to static EFs up to 50 kV/m under laboratory conditions while they indicated their perceptual impression by pressing a button under different test conditions. The average DC EF detection threshold was 45.1 kV/m. Overall, a large interindividual variability was observed. While a third of the participants were able to perceive EF strengths from 25 kV/m, another third was unable to successfully detect the maximum EF strength of 50 kV/m^4^. Building on this, in an experimental study by Kursawe et al.^6^, including 203 participants, evenly distributed across sex and age, average threshold values of 18.69 kV/m (DC), 14.16 kV/m (AC) and 6.76 kV/m (hybrid) were obtained, whereby hybrid EFs always consisted of a constant AC component of 4 kV/m and a variable DC component^6^. Significantly lower detection thresholds were found under hybrid conditions than under separate exposure to DC and AC EFs as 39% of the participants were able to successfully detect the lowest hybrid EF combination of 2 kV/m DC and 4 kV/m AC. This indicated a synergistic effect on human perception from the combination of both EF types^6^, which was verified in a follow-up study based on the results of 51 participants with very good perception of hybrid EFs^8^. Even the lowest EF strength combination of 1 kV/m DC and 1 kV/m AC was successfully perceived by one participant, which emphasizes the human sensitivity to hybrid EFs. These results of this systematic investigation confirm the initial indications of Clairmont et al.^3^, who concluded from their experiments under high-voltage overhead power lines that static EFs are perceived more strongly when 50 Hz alternating EFs are present at the same time^3^. A study by the Electric Power Research Institute (EPRI) on the perception of hybrid EFs also drew similar conclusions [^21^]. Furthermore, both the AC component as well as the DC component play a significant role in the perception of hybrid EF^8,9^.

In addition to the differences in the detection performance between different EF types, the perceptual impression of the participants also varied greatly from person to person. A slight tingling or vibration of the body hair was often described^4,5^. While DC EFs were mainly perceived in the head area (scalp hair and facial hair), AC EFs more frequently led to sensations in the arms and legs^5^. Influencing factors and possible explanations for the individual perceptual impressions could be characteristics of the hair, such as length, thickness, and humidity, but also external factors, such as relative humidity^2,5,10,6,1^. Improved detection performance of AC EFs at low relative humidity (30%) and improved detection performance of DC EFs at high relative humidity (70%) could be demonstrated under laboratory conditions^5,6^. Large interindividual variances throughout all studies on the topic of EF perception were striking.

According to the World Health Organization (WHO), exposure to EFs is not expected to have any adverse health effects ^11,12^. Static and low-frequency EFs are non-ionizing radiation and penetrate little or not at all into body tissue. Their effect is therefore limited to the body surface . To date, however, neither specific receptors nor underlying biological mechanisms for the perception of EFs have been clearly established. There are indications that receptors at the hair root, which are used for the perception of vibrations, also play a role in the perception of EFs^2,1^. In the perception of AC EFs, the movement of charges induced on the skin along the hydrated hair could lead to a mutual repulsion of the individual hair, resulting in a noticeable vibration^13^. Hair moisture in relation to relative humidity appears to be particularly decisive^10,1^. However, according to current studies, the moisture of the skin does not appear to have a direct influence on the perception of EFs^8,6^. Nevertheless, there is no study investigating the effect of individual sudomotor function measured by electrochemical skin conductance (ESC) on the EF perception. Variations in sweating intensity and in the composition of sweat on the skin and on hair exposed to an EF could also influence the individual detection performance. Odagiri-Shimizu and Shimizu^1^ found lower detection thresholds in people with longer and thicker hair in local EF exposure. Thus, individual differences in specific characteristics could be an explanation for the large variances in the detection performance of EFs. The question remains whether artificial lengthening, thickening, or smoothing of the hair, e.g., by applying mascara, could improve the individual detection performance of EFs. Earlier studies showed that the perception of EFs on hairless skin was severely limited but not excluded^2,1^. It is therefore assumed that other receptors are also involved in the perception of EFs. Correlations could be found between the ability to perceive vibrations of 31 and 63 Hz on hairless skin and the ability to detect AC and hybrid EFs^6^. This is supported by the finding that sensitivity to the oscillation of body hair is closely related to vibration perception^14^. Even if this correlation could not be replicated in a subsequent study, it strengthens the assumption of the existence of further signal transduction pathways^8^. A deeper understanding of the mechanisms of action can help to understand the distinct levels of detection performance depending on the EF type or combination. For example, Nedachi et al.^15^ recently calculated that the electrical force effect on body hair in hybrid EFs is up to three times higher than the electrical force effect in pure DC or AC EFs. This could explain the lower detection thresholds of hybrid EFs depending on the presence and the characteristics of body hair. To explain the large variances in the detection performance of EFs, to investigate the influence of individual hair characteristics and to reveal the underlying mechanisms of EF perception, there is still a great need for research on this topic.

The aim of this research project is to elucidate the influence of human hair on the perception of AC, DC, and hybrid EFs. The focus is on hair properties, their absence, and their modification by mascara or the change of environmental relative humidity.

## Methods

### Participants

A total of 30 healthy volunteers (15 female and 15 male) were included in this double-blinded interventional study. Assuming a large effect size derived from previous research^10^, we calculated a test power of 97% to find an effect depending on hair presence. The participants were aged between 18 and 35 years (average age: 25.8 years). The prerequisite for participation was that they had no previous experience of exposure to and perception of EFs. When selecting the group of participants, care was taken to include a broad spectrum of different hair characteristics (hair structure, length, color) in the study. In order to ensure a significant difference between the unshaven condition and the shaved condition, a full head of hair and a minimum hair length of 3 cm were specified. People with electronic implants or piercings, people who would describe themselves as electrohypersensitive and people with skin diseases or neurological disorders were excluded. As part of the medical assessment prior to study inclusion, a detailed medical history was taken, including substance abuse such as alcohol, drugs, or medication. It was followed by a physical examination which included parameters such as vital signs (pulse, blood pressure), signs of infection, abnormalities on the skin as well as sensory disturbances or neurological abnormalities. Female participants also underwent a urinary β-hCG test to rule out an existing pregnancy. All participants received an expense allowance totaling € 600 as well as a voucher for lunch in the in-house canteen on both test days. The project received a positive vote from the Ethics Committee of the Medical Faculty of RWTH Aachen University (EK435/21) and was preregistered with the German Clinical Trials Registry (DRKS00027400).

### Exposure laboratory

All studies on EF perception took place in the exposure laboratory at the Research Center for Bioelectromagnetic Interaction (femu), Institute for Occupational, Social and Environmental Medicine at the University Hospital RWTH Aachen. The interior dimensions of the exposure laboratory are 4 × 4 m with a ceiling height of 3 m. The participants were seated on the permanently installed chair in the middle of the otherwise empty room, while the investigator controlled the experimental investigations from an adjacent room. DC EF of up to 50 kV/m can be generated, as well as AC EF with an effective value of up to 30 kV/m. Combining both AC and DC EF in a hybrid condition, a maximum EF strength of 50 kV/m cannot be exceeded. During exposure, the participants were connected to the ground via silicone ankle electrodes, which were also used to record the leakage current. An acoustic noise signal is always played via four loudspeakers in the exposure room at a volume of 61.7 dB (A), which ensures that all audible influences are masked. The technical setup, EF distribution, calibration process, and security systems can be found in the publications by Jankowiak et al.^5^ and Krampert et al.^16^. The described exposure laboratory was used in all previous studies^5,8,9,6^.

### Test protocol

After the medical examination on the first day of the experiment (test day 1), a detailed briefing on the laboratory situation was given by the investigator. The technical background, safety equipment and the specific procedure of the detection tasks were explained, and any questions were clarified. After the required hair samples were taken on test day 1, the detection tasks began. During the exposure, the participant sat in the middle of the exposure room and followed the instructions projected onto the wall in front of them. A response box was used to indicate the current impression of perception during each trial. AC EFs (8, 16, 24, 30 kV/m) at a frequency of 50 Hz, DC EFs (14, 22, 30, 38 kV/m), as well as hybrid EFs (4.47, 8.94, 16.49, 24.33 kV/m) were tested. In hybrid EFs, a constant AC EF strength of 4 kV/m (rms value) was combined with DC EF strengths of 2, 8, 16, or 24 kV/m. In addition to the EF exposures, the same number of sham exposures (no present EF) were presented. Due to the double-blind study design and the random order of the exposures, neither the participant nor the investigator knew at which point in time an EF was present. In total, each participant took part in twelve sessions, each lasting around 15 min, which were conducted in random order. Including the medical examination, the instruction at the beginning of the day, and the breaks between the blocks, this resulted in an approximate total test duration of a maximum of 8 h.

During the entire test day, the participant’s task was to correctly recognize an EF exposure or correctly classify a sham exposure. A trial started with a 3 s lasting EF increase period. The EF then constantly remained in the room for 5 s. The question “Do you perceive an electric field?” was then displayed on the wall in front of the participant. Thereafter, participants were required to press a button on a response pad within a 4 s lasting time window referring to the following options: “yes - certain”, “yes - uncertain”, “no - uncertain” and “no - certain”. The EF was then lowered again, and the system was grounded taking 7–9 s. The next trial started without delay. In sham trials the timing was identical, but no EF was presented.

In order to investigate the influence of different environmental conditions, the relative humidity was varied within a test day. During the first six sessions in the morning, the relative humidity in the laboratory was 50%. In the afternoon, the relative humidity was reduced to 30% for half of the participants or increased to 70% for the other half. This variation was identical within one participant on both test days. The temperature was always kept constant at 22 °C.

The test procedure described was conducted twice for each participant at an interval of seven days (test day 1 and test day 2). Participants were instructed to remove all their hair from their head, arms, and face (beard hair) within 24 h before test day 2 by wet shaving. Facial hair, like eyebrows and eyelashes, were not affected by this measure. The order of the sessions, like the variation in humidity, was identical across test days, whereby the presentation of the exposure and sham sessions was randomized within the sessions to ensure a double-blind study design. On both test days, all participants were asked at which parts of the body they perceived the EF to determine differences in perception location after removing the hair.

An additional variation was added for female participants: on both test days, they were instructed to apply mascara after the first three sessions in the morning to additionally investigate any resulting changes in EF perception due to changed hair properties of eyelashes.

### Hair analyses

#### Sampling

For a detailed laboratory analysis, hair samples were taken from each participant on test day 1 immediately prior to the detection tasks. At least an amount of 150 mg of hair was cut off at various points as close as possible to the scalp. To avoid falsification of hair analyses and to keep the condition of the hair as constant as possible, the participants were instructed to wash their hair the day before and not to apply any care or styling products. In addition, about 10 hairs from both forearms were cut off preferably in full length and packed for laboratory analysis. The arm hair was also not creamed or treated beforehand.

#### Determination of hair moisture

All hair moisture measurements and microscopic analyses of participant´s hair were conducted at the Institute for Plastics Processing in Industry and Craft at RWTH Aachen University (IKV). Based on previously conducted feasibility tests, the thermogravimetric analysis (TGA) method was used for hair moisture measurements. In addition to the total percentage of water, the TGA enables to determine the external moisture (at a temperature of 65 °C) and internal moisture (at a temperature of 180 °C)^17^. The scalp hair samples of the 30 participants (three samples per participant) were conditioned in a climate chamber for 24 h prior to the TGA to reconstruct the ambient conditions in the exposure laboratory. The temperature was kept constant at approx. 22 °C (± 3 °C), while the relative humidity (30%, 50%, 70%; each with a standard deviation of ± 3%) was based on the individual environmental conditions during the detection tasks. Thus, half of the samples were conditioned with 50% and 30% relative humidity and the other half with 50% and 70% relative humidity. The samples were then hermetically sealed and reopened only shortly before the TGA measurement. In two stages, the following parameter settings were applied to the TGA for the determination of the external humidity of the scalp hair (temperature range: 30 °C to 65 °C; heating rate: 20 °C/min; isothermal holding time at 65 °C: 40 min) and for the determination of the internal humidity of the scalp hair (temperature range: 65 °C to 180 °C; heating rate: 20 °C/min; isothermal holding time at 180 °C: 30 min).

#### Microscopic analysis

Depending on the hair length, the scalp and arm hair was measured either using an analog caliper (less than 150 mm) or a steel ruler (longer than 150 mm). The hair was glued on manually under slight tension to compensate for natural curvature (e.g., curly or wavy hair). Three hairs were always measured per participant and condition. The hair thickness of scalp and arm hair was measured on the previously glued hair using a light microscope. Three positions of each hair were always measured: close to the hair root, in the middle of the hair and at the end of the hair. All hairs were measured for roughness at equivalent positions to the hair thickness measurements using a Laser Scanning Microscope (LSM). The maximum height of the roughness was calculated as the mean value of five individual roughness depths of successive individual measurement sections within the selected position. To obtain the best possible conditioning, all measurements of each hair were conducted within a maximum of 30 min after opening the sealed sample container. The hair thickness was always measured first, followed by the hair roughness and finally the hair length. For the statistical analysis, the measurements at the three positions were averaged.

### Electrochemical skin conductance (ESC)

All participants were invited to measure their individual electrochemical skin conductance (Sudoscan^®^). Twenty participants could be included (13 female). Sudoscan enables rapid, non-invasive, and precise quantification of sudomotor function mediated by small, unmyelinated nerve fibers^18,19^. Participants placed their hands and feet on stainless steel electrodes for 3 min. In addition to the real measurement, a sham measurement was also performed. By the application of low direct voltage (< 4 V), ESC of the palms (left and right) and the foot soles (left and right) was expressed in µS and compared with age-, sex-, and weight-matched controls.

### Data processing and analysis

In the present study, the signal detection theory (SDT) approach^20^ was used for the experimental design. In this way, a measure of the sensitivity d’ (d prime) can be determined for the perception of sensory stimuli. From detection task responses, four outcomes can occur: (1) EF present and detected (“hit”), (2) EF present and not detected (“miss”), (3) sham exposure and EF detected (“false alarm”) and (4) sham exposure and no EF detected (“correct rejected”). Participants’ sensitivity (d’) is determined by a z-transformation of the relative frequency of “hits” and “false alarms” and the subsequent difference between the two z-values: d’ = z(hit) - z(false alarm). Sensitivity values were calculated for each participant and each EF strength at a relative humidity of 50% and for half of the participants at 30% and 70%. Values below the detection threshold are reflected in d’ values = 0, whereas d’ values above 1 indicate successful EF detection. D’ values of 1 to 2 or 3, show good, very good, or excellent detection performance. As z-values cannot be calculated if d’ values were 0 or 1, a log-linear transformation of the “hit” and “false alarm” rates was conducted according to Hautus^21^. In hybrid EFs, combined EF strengths E_RMS_ from DC and AC were calculated:

$$\:{E}_{\mathrm{RMS}}=\sqrt{{{E}_{\mathrm{DC}}}^{2}+{{E}_{\mathrm{AC}}}^{2}}$$

where E_DC_ and E_AC_ are the respective root mean square (RMS) values of the EF strength. Detection thresholds were calculated for combined EFs, as well as for AC and DC EFs. Within the individual psychometric function, the point at which this function reaches a d’ of 1 is thus considered to be the threshold value regarding the EF detection. This was only possible if the individual course was plausible, i.e., showing an increase in d′ with increasing EF strength. For example, if a d′ ≥ 1 was found at the lowest EF strength, but a d′ < 1 was observed at a higher EF strength, the course was classified as implausible. Furthermore, if the participants were unable to detect EFs, e.g., at the highest EF strength, no detection threshold could be calculated.

For the statistical evaluation of the threshold values, repeated measures analyses of variance (rm ANOVAs) were calculated for each EF type (AC, DC, hybrid) with the factor *test day* (1, 2). For the specific examination of the influence of relative humidity, rm ANOVAs were calculated for each EF type (AC, DC, hybrid) with the factors *EF strength* (DC: 14, 22, 30, 38 kV/m; AC: 8, 16, 24, 30 kV/m; hybrid: 4.47, 8.94, 16.49, 24.33 kV/m), *test day* (1, 2), and *relative humidity* (50%, 30%, or 50%, 70%). As the relative humidity was examined in two subgroups (30% and 50%, as well as 50% and 70%), the number of possible participants was halved (*n* = 15). To investigate the influence of mascara during a relative humidity of 50%, an rm ANOVA was calculated for each EF type with the factors *EF strength* (DC: 14, 22, 30, 38 kV/m; AC: 8, 16, 24, 30 kV/m; hybrid: 4.47, 8.94, 16.49, 24.33 kV/m), *test day* (1, 2) and *mascara* (with, without) exclusively including all female participants (*n* = 15). For reasons of relevance, only statistically significant results were reported with regard to *mascara*. An α-level of *p* = 0.05 was accepted for statistical significance, and partial eta-squared values (η_p_²) were used to estimate effect sizes. When sphericity could not be accepted, corrected Greenhouse-Geisser^22^ significance values were reported with uncorrected degrees of freedom.

Correlational analyses were performed to elucidate the relationships between the parameters of the microscopic hair analyses (length, thickness, roughness) and the detection thresholds. Relations between TGA and detection thresholds were also considered correlatively. Only the detection thresholds from test day 1 (with hair) were used for the correlation analyses, as the influence of the hair with all its characteristics is given here. ESC values were also correlated with AC, DC, and hybrid thresholds. Therefore, ESC values of feet and palms as well as detection thresholds of both test days were averaged, respectively. The Pearson coefficient *r* was calculated for the detection thresholds across all test conditions. A coefficient of *r* = 0 shows no correlation, while a value of *r* = 1 shows a perfect correlation. In order to relativize the influence of the number of analyses on the alpha error, a Bonferroni correction was applied for each set of correlation analyses.

The evaluation of the survey data with regard to the place of perception was evaluated numerically and descriptively. Clusters were formed for the scalp, arms, and face regions. Scalp included mentions such as scalp hair, hairline, or head. The arm region included mentions such as upper arm, forearm, fingers, or palms. The face region included mentions such as forehead, temple, cheek, mouth area, beard, and chin. Eyebrows, ears, and eyelashes were considered individually.

## Results

### Effects of relative humidity and hair removal

**Fig. 1:**
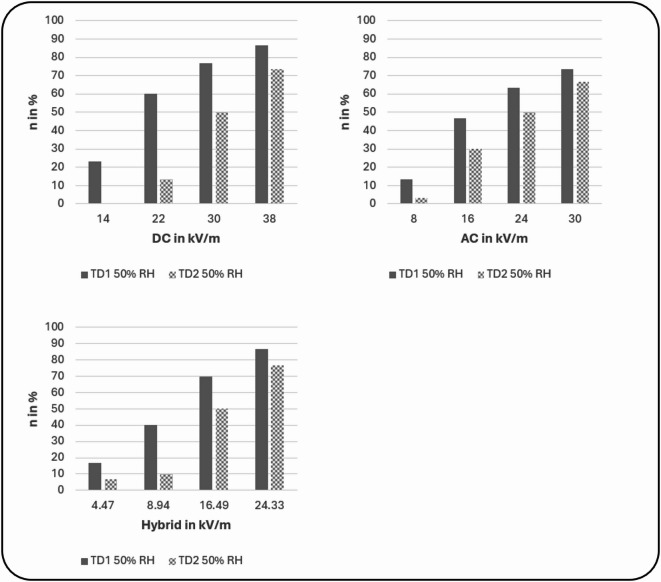
Percentage of participants who successfully perceived a certain EF strength (d’ ≥ 1) for DC, AC, and hybrid EF, for each test day (test day 1 with hair and test day 2 after hair removal) at a relative humidity of 50%.

On a descriptive level, the number of participants who showed a successful detection (d’ ≥ 1) for a specific EF type and strength increased with increasing EF intensity (see Fig. 1). At a relative humidity of 50%, each EF strength (four per EF type) was perceptible at least by some participants on test day 1. D-Prime values also increased with increasing EF strengths. However, after hair removal on test day 2, the number of participants showing a successful detection for a specific EF strength decreased remarkably.

**Fig. 2:**
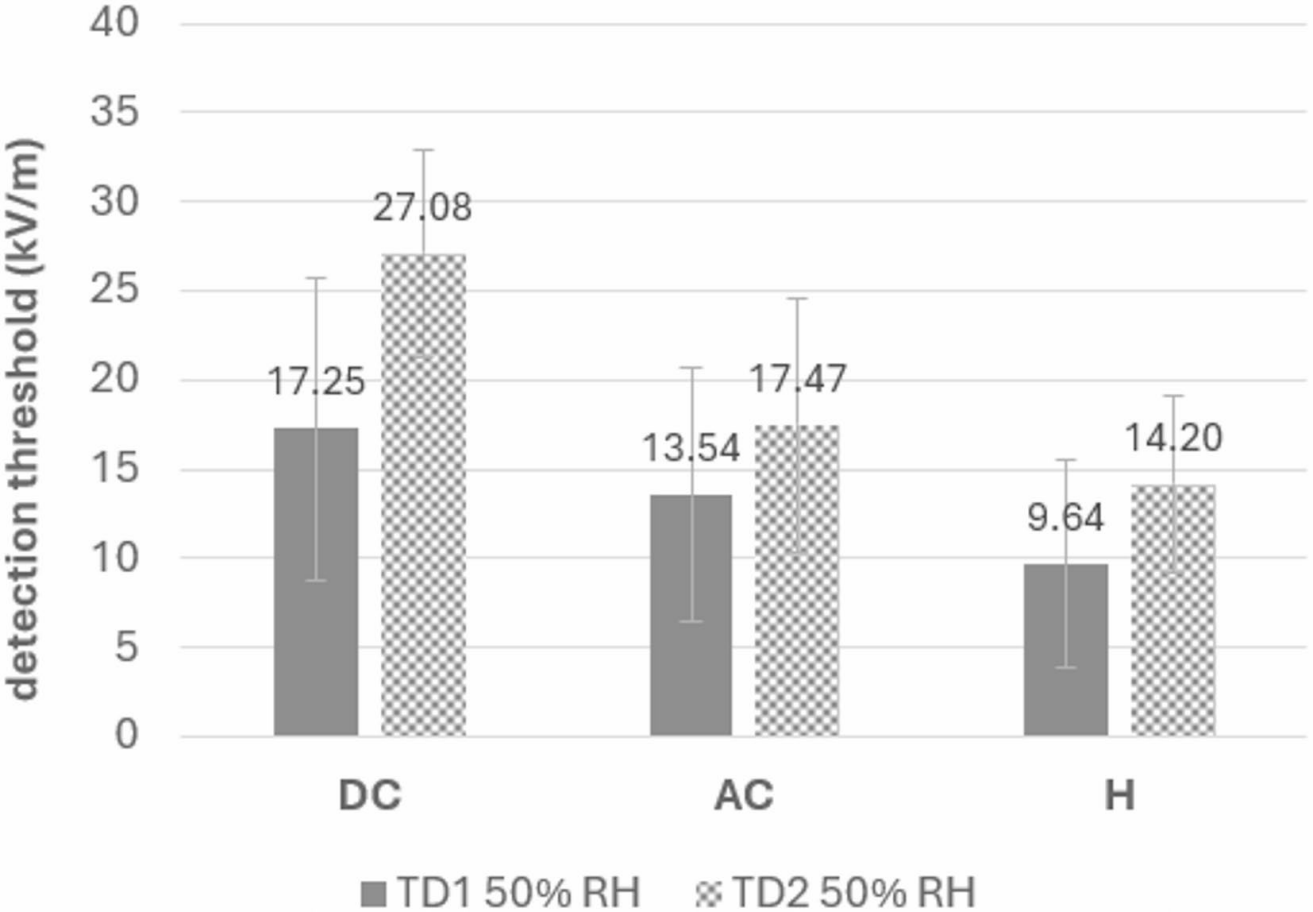
Detection thresholds for DC, AC, and hybrid EFs at a relative humidity of 50%. The number of participants included were 21, 16, and 21 for DC, AC, and hybrid condition. Only participants for whom detection thresholds could be calculated for both test days were included. Bars represent standard deviations.

The detection threshold increased after hair removal in all EF types, as well (see Fig. 2). The one-factorial rm ANOVAs on detection thresholds for DC (*n* = 21), AC (*n* = 16), and hybrid EF (*n* = 21) with the factor *test day* (1, 2) showed a significant main effect for DC and hybrid EFs [(*F*(1, 20) = 16.46, *p* < 0.001, η_p_^2^ = 0.45), (*F*(1, 20) = 20.63, *p* < 0.001, η_p_^2^ = 0.51)], but not for AC (*p* ≥ 0.13).

**Fig. 3:**
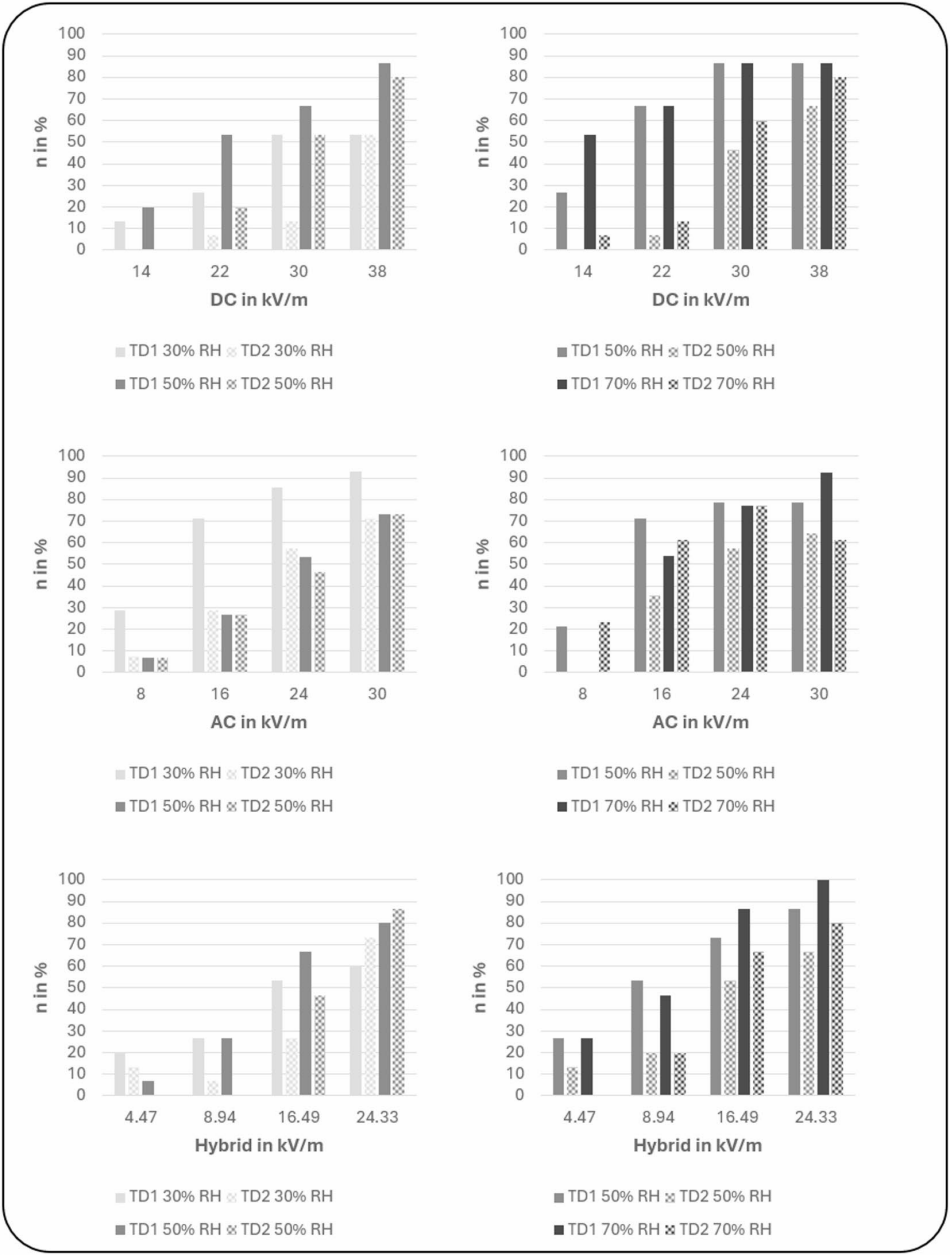
Percentage of participants who successfully perceived the applied EF strengths (d’ ≥ 1) for DC, AC and hybrid EFs divided into the two groups of participants who experienced either 30% and 50% or 50% and 70% relative humidity, and both test days (test day 1 with hair and test day 2 with removed hair). Within all diagrams, the same participants are compared with each other in different conditions (*n* = 15).

Concerning the effect of relative humidity on EF perception (see Fig. 3), in DC EF, the number of participants showing a successful detection increased, or in some cases remained constant, with increasing relative humidity. In AC EF, a reversed pattern was found as in most cases, the number of participants showing a successful detection decreased with higher relative humidity. In hybrid EFs, more participants showed a successful detection in low relative humidity when low EFs (4.47 kV/m and 8.94 kV/m) were presented, whereas in high EFs (16.49 kV/m and 24.33 kV/m) more participants successfully perceived the EF in high relative humidity. In the analyses of the subgroups with 30% and 50% relative humidity, significant main effects were found for relative humidity for DC and hybrid [(*F*(1, 14) = 23.1, *p* < 0.001, η_p_^2^ = 0.62), (*F*(1, 14) = 5.99, *p* < 0.05, η_p_^2^ = 0.3)], but not for AC (*p* ≥ 0.14). In the subgroup experiencing 50% and 70% relative humidity, a significant effect was observed for DC and AC [(*F*(1, 14) = 5.28, *p* < 0.05, η_p_^2^ = 0.27), (*F*(1, 12) = 13.01, *p* < 0.05, η_p_^2^ = 0.52)], but not for hybrid EFs (*p* ≥ 0.07).

### Influence of electrochemical skin conductance and hair characteristics

ESC values varied between 60.2 and 93.6 µS. On a numerical level, increasing ESC values were associated with increasing AC detection thresholds whereas in DC and hybrid conditions, increasing ESC values came along with decreasing detection thresholds. On a statistical level, no significant correlations were found between AC, DC, and hybrid detection thresholds and ESC values of palms and foot soles (*p* ≥ 0.08).

**Fig. 4:**
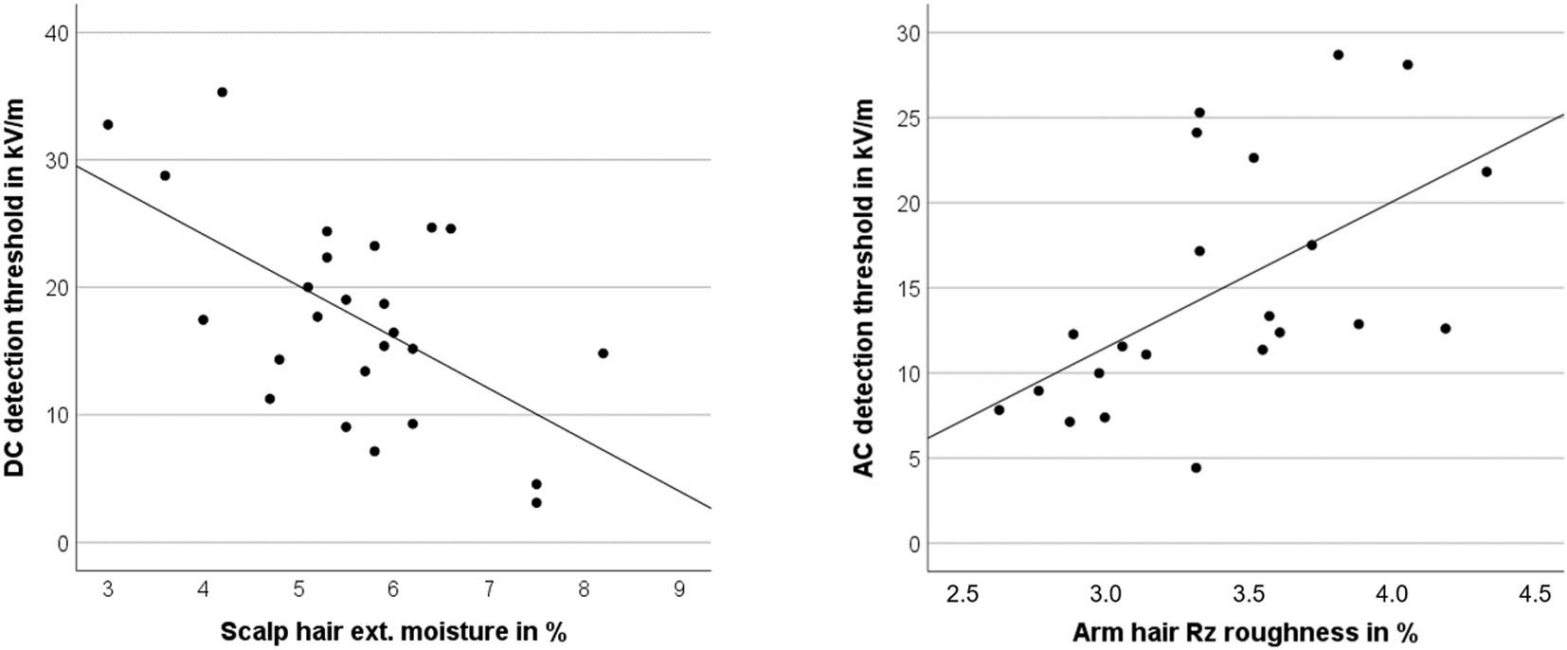
Left: Correlation of the DC detection thresholds on test day 1 with the external humidity of the scalp hair (KH_ExtHum) for *n* = 25 participants in 50% relative humidity. Right: Correlation of the AC detection thresholds on test day 1 with the roughness of the arm hair (AH_Rz) for *n* = 22 participants in 50% relative humidity.

The total moisture content of the hair samples conditioned at 50% relative humidity varied between 6.3 and 12.5%. The measurements of the physical hair properties showed also large variations in the length, the thickness and the roughness of scalp and arm hair. A statistically significant correlation was found between the DC detection threshold and the external humidity of the scalp hair (*r* = -0.59, *p* = 0.016) and between the AC detection thresholds and the roughness (Rz) of the arm hair (*r* = 0.56, *p* = 0.014). The correlations are shown in Fig. 4.

**Fig. 5:**
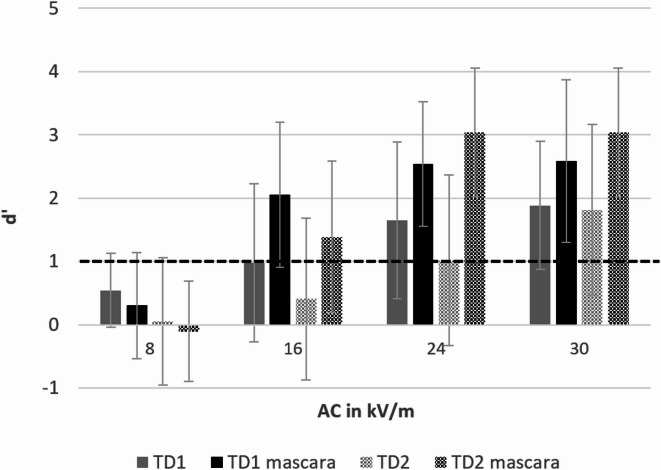
Sensitivity d’ for all presented AC EFs at a relative humidity of 50%. Only female participants (n = 15) were instructed to apply mascara on both test days (TD1 and TD2) after the first three sessions. Bars reflect standard deviations. The dashed line represents a d’ of 1 indicating a successful detection.

To investigate the influence of mascara under 50% relative humidity in female participants (*n* = 15), a rm ANOVA on detection thresholds with the factors *EF strength* (DC: 14, 22, 30, 38 kV/m; AC: 8, 16, 24, 30 kV/m; hybrid: 4.47, 8.94, 16.49, 24.33 kV/m), *test day* (1, 2) and *mascara* (with, without)) showed no main effect for *mascara* (*p* ≥ 0.78) in DC EFs. However, there was a statistically significant influence in AC and hybrid EFs [(*F*(1, 12) = 5.37, *p* = 0.039, η_p_^2^ = 0.31), (*F*(1, 14) = 7.78, *p* = 0.014, η_p_^2^ = 0.36)]. Significant interactions with *mascara* were not found (all *p* ≥ 0.16). Thus, the sensitivity values in hybrid and especially in AC EFs (see Fig. 5) were remarkably higher after mascara was applied.

### Perception location

**Fig. 6:**
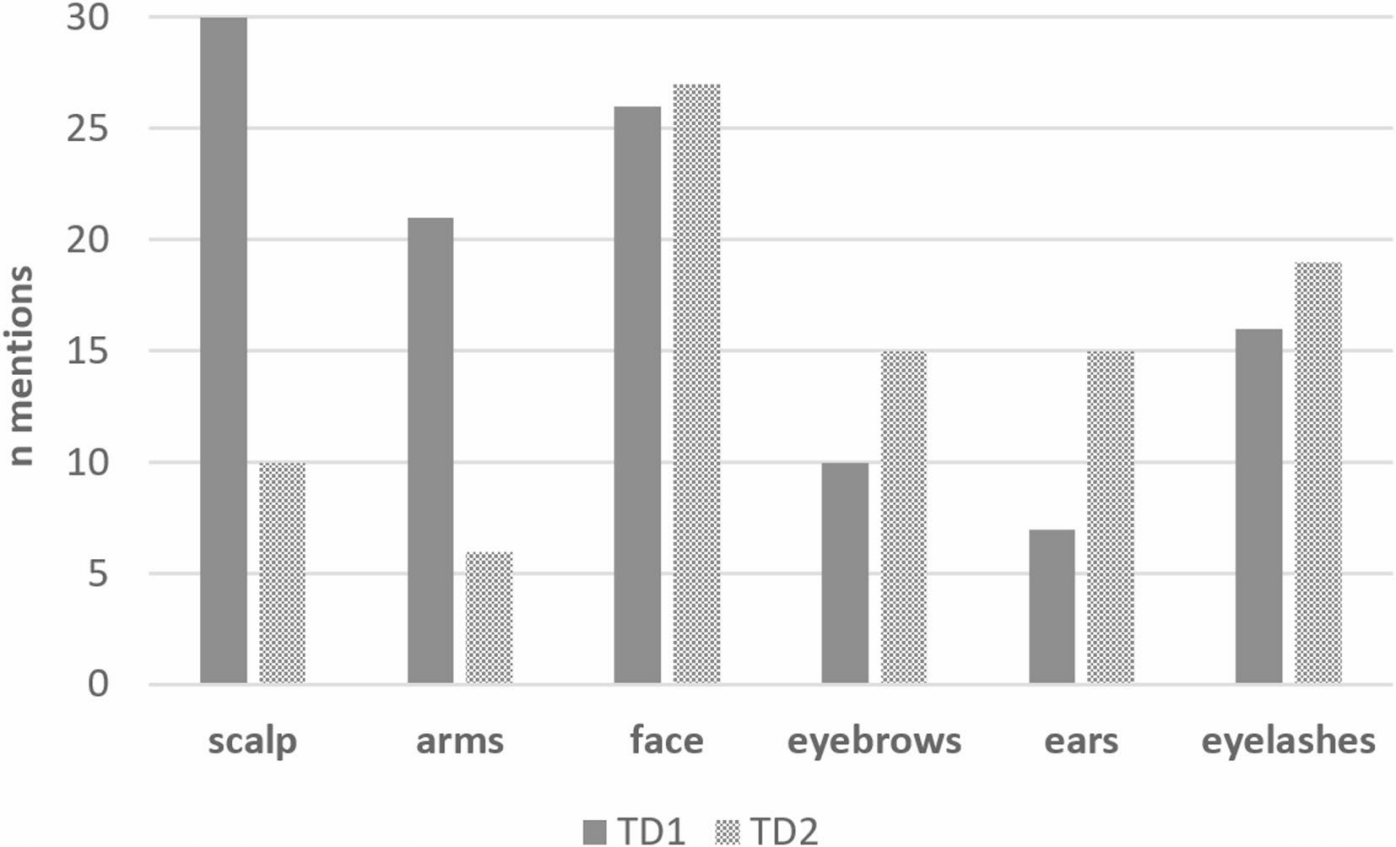
Number of participants (*n* = 30), who mentioned the perception locations on test day 1 and 2. Multiple answers were possible.

Regardless of the EF type, all participants located a perception at the scalp on test day 1 (see Fig. 6). More than two-thirds also located a perception on their arms. On test day 2, the number of mentions at the scalp and on the arms decreased remarkably. Facial hair, like eyebrows or eyelashes, were not removed on test day 2 and the number of mentions increased in these areas.

## Discussion

The aim of this research project was to identify factors influencing EF perception with focus on human hair. Hair properties, the effect of their absence, and their modification by environmental influences, like relative humidity or applied mascara were investigated. Results showed that the ability to perceive EFs decreased enormously when hair was removed. Correlations were found between the ability to perceive EFs and the structural properties and humidity of the hair. Environmental factors, like relative humidity and the application of mascara, directly influenced the EF detection performance.

Concerning the detection thresholds and the effects of changes in relative humidity, the results of the present study fit in well with already known patterns of results^5,6^. While high relative humidity improved the detection performance of DC EFs, low relative humidity improved the detection performance of AC EFs. In hybrid EFs, the influence of relative humidity on the detection performance depended on the EF strength. In low hybrid EFs, low relative humidity enhanced the detection performance, whereas in high EFs, low relative humidity impaired the detection performance. For each EF type, Kursawe et al.^6^ showed similar effects of relative humidity. In general, this opposite effect of relative humidity on the detection performance of different EF types was also evident on test day 2, at least in AC and hybrid EFs, showing its robustness. Overall, the data of the present study show a large interindividual variance, which is already known from previous studies^5,8,9,6^. The aim of the project described here was to clarify parts of this variance.

The removal of head and arm hair led to a remarkable decrease in the number of participants showing a successful detection for DC, AC, and hybrid EFs. A relatively small number of participants (3%) perceived AC EFs of 8 kV/m and no participant perceived DC EFs of 14 kV/m after hair removal on test day 2. This is also reflected by an associated increase in the detection thresholds, which was particularly evident for DC EFs, where increases in the detection threshold of 10 kV/m were observed. The results are consistent with previous findings on local exposure to DC EFs. Odagiri-Shimizu and Shimizu^1^ showed that removal of hair in the area of the forearm led to significant limitations in EF perception. The influence of hairiness on the perception of DC EFs was found in all variations of relative humidity. Regarding AC EFs, it has previously been shown that the removal of hair during local exposure to AC EFs led to significant impairments in EF detection^2^. These results confirm present AC EF findings in low and 50% relative humidity enabling a transfer to whole-body testing. Interestingly, in high relative humidity, the removal of hair seemed to have no consistent reducing effect on the detection performance possibly indicating a humidity-dependent mechanism in perception: Without hair, AC EF detection performance is improved by high relative humidity, whereas the known force effect on hair in AC EFs is favored by a reduction in relative humidity^6,13^. The increased conductivity caused by high relative humidity could therefore also improve the detection of AC EFs, especially when no hair is present^6,13^.

Results from Sudoscan confirmed that a healthy group of participants was included without any nerve-related sudomotor dysfunction, potentially confounding our results^19^. In general, sweat function on the palms and soles was not related to EF detection performance. Accordingly, participants with a very good ability to detect EFs do not sweat more on their palms and soles than other participants. However, low ESC values seem to facilitate AC EF detection fitting in with the effects of low relative humidity on the AC EF detection previously discussed^6^. More research on the individual amount and composition of sweat, especially on hairy skin, is needed. Correlations between parameters of head hair and DC EF perception and parameters of arm hair and AC EF perception seem plausible, as it has already been shown that arms are prominent in AC EF perception, whereas the head area is prominent in DC EF perception^5,6^. Neither the thickness nor the length of the hair seems to influence the ability to detect the EFs. Nevertheless, hair roughness as a structural parameter thus appears to have an influence on the force effect of the AC EF on the arm hair. Smoother hair seems to facilitate AC EF perception. This finding is also supported by the effect of mascara. An improved detection performance in AC EFs with applied mascara indicates that hair which has been smoothed in terms of roughness vibrates better or that the movement of hair can be better perceived. The increased weight of the hair due to the mascara application may also play a role. Furthermore, higher hair moisture in the head area is associated with a lower detection threshold for DC EFs. This supports earlier explanatory models that assume an advantage of conductive processes in higher relative humidity and thus an advantage for the perception of DC EFs^6,13^. Overall, interindividual variances in EF performance could be partially explained by different hair characteristics, especially concerning hair roughness and head hair moisture.

Perception locations changed markedly from test day 1 to test day 2. Whereas the number of mentions at the scalp and on the arms decreased remarkably on test day 2, perceptions in the face, the eyebrows, ears, and eyelashes increased. Shaving the scalp and arms therefore led to reduced perception in these areas of the body, which is consistent with the result of reduced EF perception on test day 2. However, a third of participants still perceived an EF on the shaved scalp. These findings support earlier studies showing that the perception of EFs on hairless skin is severely limited but not excluded^2,1^. This could be explained by the assumption that not only receptors indicating hair movement, but also other receptors are involved in the EF perception. On test day 2, an increased number of perception mentions was found on hairy body areas such as the face (including forehead, temple, cheek, mouth area, beard, chin), eyebrows, ears, and eyelashes, which were not shaved. This could be explained by the loss of a strong sense of perception at the scalp or on the arms, forcing the participants to focus on different body areas, maybe on those with particularly fine hair showing a lower sense of perception.

### Limitations

When considering the effects of relative humidity, two subgroups were formed. Therefore, the comparison of the effects between the groups with 30% and 50% relative humidity and 50% and 70% relative humidity is a between-person comparison. Differences, e.g., with regard to the different influence of humidity on the AC threshold, could therefore also have group-specific causes.

The removal of head and arm hair was carefully checked. Care was also taken to ensure that hair in the finger area was removed as well. Nevertheless, small inaccuracies in shaving or minor shaving injuries cannot be ruled out completely. It should also be noted that the hair was not completely removed, but only clean-shaven. This means that the hair root and the part of the hair located in the skin were still present. This fact could explain why it was possible to perceive the EF even on shaved parts of the body. Nevertheless, the results may also indicate that in addition to perception via the hair, there are other mechanisms of action and receptors that we can use to detect an EF.

## Conclusions

The replication of the effect of relative humidity on the perception of various EF types underscores the robustness of results in EF detection research. The influence of hair on the head and arms on the ability to perceive EFs is clearly emphasized. It was shown that the removal of hair is associated with a strong loss of detection performance. At the same time, if hair is present, its characteristics also have an influence on EF detection performance. Head hair moisture in DC EFs and hair roughness in AC EF perception influence the detection thresholds. The results complement the known models regarding EF perception and their extensions^6,13^. Furthermore, the results help to understand how EFs are perceived by humans. Further research focusing on the influence of fine hairs on the ears, cheeks, and eyelashes is needed. It remains to be investigated whether complete hair removal could eliminate the ability to perceive EFs.

## Data Availability

The datasets used and analyzed during the current study are available from the corresponding author on reasonable request.

## Abbreviations

EF: electric field
AC: alternating current
DC: direct current
ESC: electrochemical skin conductance
SDT: signal detection theory
TD1: test day 1
TD2: test day 2
RH: relative humidity
